# Development of AI-based dopamine transporter (DAT) image generation technique using early phase [^18^F]-FP-CIT PET imaging

**DOI:** 10.1371/journal.pone.0349375

**Published:** 2026-05-14

**Authors:** Changhwan Sung, Jungsu S. Oh, Sun Young Chae, Dong Yun Lee, Minyoung Oh, Sang Ju Lee, Seung Jun Oh, Sungyang Jo, Sun Ju Chung, Jae Seung Kim

**Affiliations:** 1 Department of Nuclear Medicine, Asan Medical Center, University of Ulsan College of Medicine, Seoul, Republic of Korea; 2 Department of Nuclear Medicine, Uijeongbu Eulji Medical Center, Eulji University School of Medicine, Uijeongbu-si, Republic of Korea; 3 Department of Neurology, Asan Medical Center, University of Ulsan College of Medicine, Seoul, Republic of Korea; Banner Alzheimer’s Institute, UNITED STATES OF AMERICA

## Abstract

**Objectives:**

To develop and validate a deep learning-based model capable of generating dopamine transporter (DAT) images from early-phase [^18^F]-FP-CIT positron emission tomography (PET) imaging.

**Materials and methods:**

Conditional generative adversarial network was trained using 477 dual-phase [¹⁸F]-FP-CIT PET scans acquired with a conventional PET system. The model generated delayed-phase images from early-phase dynamic scans (30–40 min post-injection), using five adjacent axial slices to predict the central delayed-phase slice. The model was evaluated using an internal validation set from the same scanner and an independent prospective validation set from a digital PET system. Striatal binding ratios (SNBR) and inter-subregional ratios were compared using Pearson’s correlation and receiver operating characteristic-area under the curve (AUC) analyses.

**Results:**

Generated images showed high similarity to real delayed images. SNBRs for the whole striatum correlated strongly between real and generated images (R = 0.93 and 0.90 for internal and independent sets, respectively). Diagnostic performance was comparable with the highest AUC observed in the posterior putamen (real vs generated: 1.00 vs 0.98, p > 0.2). Visual assessments revealed no uninterpretable images, and interpretability did not differ significantly. Diagnostic accuracy of generated images was comparable to that of real images in the internal validation set for detection of abnormality (p = 0.453) and degenerative parkinsonism (DP) (p = 1.000). In the independent validation set, DP detection remained comparable (p = 0.25), whereas real images demonstrated significantly higher accuracy for abnormality detection (p < 0.001).

**Conclusion:**

Deep learning-based model generated DAT images achieve satisfactory performance in quantitative and visual assessments across internal and independent validation sets.

## Introduction

[^18^F]-FP-CIT selectively binds to the dopamine transporter (DAT) located on the terminals of dopaminergic neurons, enabling the visualization of dopaminergic neuronal degeneration. This radiotracer has been extensively employed in diagnosing Parkinson’s disease (PD) [1–6]. After injecting the radiotracer, striatal-to-background contrast increases progressively, reaching a plateau at approximately 2–3 h. Although studies have explored the optimal imaging time, acquiring delayed images at 90–120 min post-injection has no significant effect on diagnostic accuracy [7]. However, owing to patient conditions and hemodynamic variability, the 3-h post-injection image is considered the most stable [7,8]. Nevertheless, patients undergoing [^18^F]-FP-CIT DAT scanning are often older aged or have movement disorders and psychosocial comorbidities, making prolonged waiting periods challenging. Additionally, extended imaging protocols potentially increase the risk of radiation exposure for healthcare personnel and others in the hospital environment during the 3-h post-injection interval.

Artificial intelligence (AI) has been widely explored in medical imaging applications. Recently, AI has been applied to image reconstruction [9], automated quantification [10,11], and image synthesis [12–15]. Conditional image-to-image translation networks, particularly those using convolutional neural networks (CNN) and adversarial training, have been successfully applied to MR-to-CT conversion, positron emission tomography (PET)-to-MRI synthesis, and contrast-enhanced CT generation [16–18]. In nuclear medicine [19,20], deep learning has shown potential in generating full-dose PET images from low-dose inputs [21,22] or synthesizing delayed-phase images from early or dynamic frames in amyloid PET [23].

Given the potential of AI in imaging applications, this study investigated the feasibility of early-to-delayed image translation, specifically the synthesis of delayed-phase images from early-phase dynamic images. The early-phase (approximately 0–10 min post-injection) reflects cerebral perfusion and serves as a surrogate for [^18^F]-FDG uptake. However, this early-phase image is perfusion-dominant and may not adequately capture delayed-phase features, which primarily reflect DAT binding in the striatum (approximately 90 min to 4 h post-injection). The 30–40 min post-injection window may represent a more optimal early-phase for image synthesis.

A deep learning framework based on conditional generative adversarial networks (cGANs) is proposed in this study to generate high-fidelity delayed-phase [^18^F]-FP-CIT PET images. The proposed model employed a two-dimensional (2D) multi-slice U-Net-based generator that captures both local and contextual spatial information across axial slices, enabling accurate mapping between early-phase PET and both DAT binding and structural representations. This approach potentially reduces acquisition times, enhances the diagnostic completeness of incomplete datasets, and enhances patient and clinical workflow efficiency. Model performance was evaluated using image similarity metrics and task-specific assessments, including a comparison of quantitative striatal uptake values between real and synthesized delayed-phase [^18^F]-FP-CIT PET images.

## Methods

### Study population and [^18^F]-FP-CIT PET/CT imaging

This study included two cohorts. The first dataset, used for model training and internal validation, comprised patients who underwent [^18^F]-FP-CIT PET for parkinsonism evaluation between April 1, 2018 and November 30, 2018 using a conventional PET/CT scanner (Biograph Truepoint 40, Siemens, Knoxville, TN, USA) which offers an in-plane spatial resolution of 2.0 mm full width at half maximum (FWHM) at the center of the field of view (FOV). Early-phase imaging was acquired from 30–40 min post-injection (10 min acquisition), and delayed-phase imaging was performed 180 min after intravenous administration of 185 MBq of [¹⁸F]FP-CIT. After a spiral-mode CT scan of the brain (performed at 120 kVp and 380 mA with the CARE Dose 4D system), PET emission data were collected in 3D mode with a transaxial FOV of 300 mm. Attenuation correction was performed using low-dose CT data, and PET images were reconstructed using an ordered subset expectation maximization (OSEM) algorithm with point spread function (PSF) modelling (TrueX algorithm) using six iterations and 16 subsets, along with post-reconstruction smoothing (also known as all-pass filter). The reconstructed images used a 336 × 336 matrix with a voxel size of 0.89 × 0.89 × 1.5 mm.

The independent validation cohort was prospectively collected between June 1, 2022 and December 30, 2022, which included both healthy volunteers and patients with PD. Imaging was performed using a digital PET/CT scanner (Biograph Vision 600, Siemens), which offers an in-plane spatial resolution of less than 2.0 mm FWHM at the center of the FOV, acquiring both early-phase and delayed-phase images at the same time window and transaxial FOV with a conventional PET scanner. Digital PET images were reconstructed using an OSEM algorithm with PSF modelling (TrueX), time-of-flight methods, and CT-based attenuation correction. Reconstruction parameters included eight iterations, five subsets, a Gaussian post-smoothing filter of 2.0 mm FWHM, and 440 × 440 matrices, resulting in a voxel size of 0.68 × 0.68 × 1.5 mm. Side-by-side comparison of the acquisition protocols and reconstruction parameters for both conventional and digital PET/CT system is provided in S1 Table.

A board-certified neurologist sub-specialized in movement disorders established final diagnosis for all participants in both cohorts following a minimum follow-up of 2 years. This study was approved by the Institutional Review Board (IRB No. 2022−0682, No. 2025−0650) and conducted in accordance with the Declaration of Helsinki and institutional guidelines. Informed consent was waived by the Ethics Committee for the retrospective cohort used in model training, and written informed consent was obtained from all participants in the prospective validation cohort.

### Image processing and the delayed image generation model

To capture tracer kinetics while maintaining an adequate signal-to-noise ratio, we reconstructed early-phase images into 2-min frames from the list-mode 10 min data. All scans were spatially realigned (between early-phase frames) or co-registered (early-to-delayed phase images) and resampled into an isotropic voxel spacing of 2 × 2 × 2 mm^3^. Early-phase input images were scaled to a range [−1, 1]. However, using min-max normalization, delay-phase standardized uptake values ratio (SUVR) label images with an upper limit of SUVR 10 were scaled to a range [−1, 1]. Consequently, delay-phased PET image intensities were normalized to SUVR using the volume of interest (VOI) of the occipital lobe, which was generated through a deep learning-based method adapted from a previous study employing a simple 2D U-NET architecture [24].

**Fig 1:**
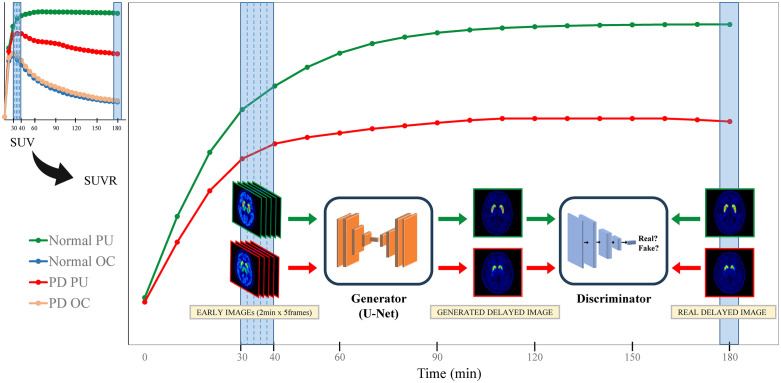
Schematic diagram illustrating the concept of the generation model by integrating with [^18^F]-FP-CIT TAC. At the top left, a small TAC graph shows the SUV over time in the defined VOIs from the PU and OC for both normal participants and patients with PD. When the OC is used as the reference region, the resulting SUVRs over time are plotted in the bottom right graph, which demonstrates a growing difference between normal and PD groups as time progresses. After the peak uptake in the PU, a series of five frames of early PET imaging (each 2 min in duration) acquired between 30 and 40 min post-injection was used as input to a generator in a cGAN model. The generator was trained to produce delayed images that resembled the real delayed PET image acquired at 180 min post-injection. A discriminator simultaneously evaluated whether the generated delayed image could be distinguished from the real delayed image, allowing the generator to improve its output through adversarial training. Abbreviations: TAC, time-activity curve; SUV, specific uptake value; VOI, volumes of interest; PU, putamen; OC, occipital cortex; PD, Parkinson’s disease; SUVR, standardized uptake value ratio; PET, positron emission tomography; cGAN, conditional generative adversarial network.

A cGAN was applied to synthesize delayed-phase PET images from early-phase inputs. The generator employed a 2D multi-slice U-NET architecture, conditioning on a stack of five consecutive axial slices from the early-phase PET volume to generate the corresponding central delayed-phase slice [25]. To include only useful slices covering the brain parenchyma, a 15 cm axial FOV was selected from the skull vertex downward, and then the top three and bottom two slices were removed from this because these slices were likely to contain minimal anatomical information and could introduce noise during the training. This design provides spatial context along the superior-inferior axis. The generator features an encoder-decoder structure with symmetric layers, skip connections between the encoder and decoder, and instance normalization following each convolutional block. Leaky-ReLU activations (slope = 0.2) were applied throughout both the encoder and decoder networks. At the bottleneck, feature maps with dimensions smaller than 64 × 64 underwent dropout regularization (rate = 0.5). The final output layer used a hyperbolic tangent (tanh) activation to produce normalized output in the range [−1, 1]. The discriminator, implemented as a CNN-based encoder, classified the input image pair (early-phase slice stack and real or generated delayed-phase slice) as real or fake (Fig 1, S1 Fig).

To incorporate three-dimensional spatial context while maintaining the efficiency of 2D convolutional networks, we adopted a multi-slice 2D input (2.5D) strategy. The model takes five consecutive axial slices from the early-phase PET scan and predicts the central slice of the delayed-phase PET scan. Instead of patch-based training, full-slice inputs were used to preserve global spatial context across the field of view, which is important for modeling spatially distributed tracer uptake patterns. The number of input slices (five) was empirically determined to provide sufficient superior–inferior anatomical context while maintaining manageable GPU memory usage.

To mitigate scanner-dependent variability between the conventional PET system (Biograph TruePoint 40) and the digital PET system (Biograph Vision 600), additional smoothed images were generated using Gaussian kernels with full width at half maximum (FWHM) of 4 mm and 8 mm and stacked with the original reconstruction as multi-channel inputs (original, 4 mm-, and 8 mm-smoothed). Because both scanners employ point spread function (PSF)-based reconstruction with resolution recovery, the effective spatial resolution is already almost compensated between systems (approximately 2 mm FWHM or less), thereby reducing the need for explicit resolution harmonization. Accordingly, the multi-scale inputs mainly provide resolution-context information to improve the robustness of the cGAN model against subtle scanner-dependent variations across hardware generations.

The generator was trained using combined adversarial and reconstruction (or fidelity) losses. The total loss was defined as follows.


$$\begin{array}{c} {\text{L}}_{total}\;=\lambda_{GAN}{\text{L}}_{GAN}\;+\lambda_{L1}{\text{L}}_{L1} \end{array}$$

where *L*_*GAN*_ represents the adversarial loss per standard cGAN formulation, and L_L1_ denotes the pixel-wise mean absolute error between generated and real delayed-phase slices. The weighting factors were empirically set to λ_GAN_ = 1.0 and λ_L1_ = 100.0 following established practice.

The model was trained using the Adam optimizer with a learning rate of 2 × 10^−4^. Mini-batch training was performed using a batch size of one over 200 epochs. Data augmentation included random rotations (±10°), shears, and scaling to enhance robustness. During inference, the delayed-phase volume was reconstructed by sliding the generator along the axial axis with overlapping input slice stacks. Predicted slices were then concatenated to form the final 3D volume.

### Quantitative analysis of image quality and diagnostic performance of the generated delayed images

To assess the similarity between real and generated delayed images, we calculated peak signal-to-noise ratio (PSNR), structural similarity index measure (SSIM), and root mean square error (RMSE).

PSNR, which evaluates voxel-wise intensity similarity, is defined as follows.


$$\begin{array}{c} PSNR\;=\text{10 }{log}_{\text{1}\text{0}}\left(\frac{{Max}^{\text{2}}}{MSE}\right) \end{array}$$

where Max is the maximum voxel intensity, and MSE stands for mean squared error between real and generated delayed images. SSIM quantifies perceptual and structural similarity, accounting for luminance, contrast, and spatial structures. It was computed on a per-slice basis using a 2D sliding window and averaged across the volume. The formula for SSIM is as follows.


$$\begin{array}{c} SSIM=\;\frac{\left(\text{2}\mu_{x}\mu_{y}+{\text{c}}_{1}\right)+(\text{2}\sigma_{xy}+{\text{c}}_{2})}{\left({\mu_{x}}^{2}{\mu_{y}}^{2}+{\text{c}}_{1}\right)\left({\sigma_{x}}^{2}{\sigma_{y}}^{2}+{\text{c}}_{1}\right)} \end{array}$$

where μ_x_, μ_y,_ σ_x_, σ_x_, and σ_xy_ represent local means, variances, and covariance of the real and generated delayed images, respectively. Additionally, RMSE was calculated to quantify voxel-wise agreement between real and generated delayed images.

For quantitative analysis, we used a deep learning-based automated method to define the VOI and calculate the specific-to-non-specific binding ratio (SNBR) in the striatum, using the occipital lobe as the reference region. In addition to the whole striatum, subregional SNBR values, including putamen (P), anterior putamen (AP), posterior putamen (PP), caudate nucleus (CA), and ventral striatum (VS), were measured as described in a previous study [26]. Correlation analysis was conducted to compare subregional SNBRs between real and generated delayed images. To evaluate diagnostic performance for degenerative parkinsonism (DP), we conducted receiver operating characteristic (ROC) curve analysis, incorporating the six striatal SNBR values and four inter-subregional ratios (ISR): CA/P, PP/AP, C/VS, and P/VS [26]. Furthermore, permutation tests were performed to compare the area under the curve (AUC) values for striatal SNBR and ISR between real and generated delayed-phase images.

### Visual analysis of the generated delayed images

Generated delayed-phase images from the cGAN model were randomly mixed with real delayed-phase images from the internal and independent validation sets and blindly reviewed by three board-certified nuclear medicine specialists with 28, 18, and 15 years of experience, respectively.

Image quality was categorized as good if no abnormalities related to noise (increased irregular background activity) or motion (structural distortion and/or abnormal striatal morphology) were observed; fair if minor artefacts were present, but the image remained interpretable; and poor if images were deemed uninterpretable owing to significant noise and/or motion artefacts. Interpretability for generated and real delayed images was then compared.

Uninterpretable images were excluded from the diagnostic performance analysis. The remaining images were classified into three categories: (1) normal DAT binding pattern, characterized by the absence of focal or diffuse uptake reduction; (2) typical DP pattern, characterized by abnormalities including PD, multiple system atrophy, progressive supranuclear palsy, and corticobasal degeneration [5]; and (3) non-DP (nonDP) pattern, indicating abnormal findings inconsistent with the typical DP pattern. Evaluations from the three readers were consolidated into a consensus classification based on visual interpretation. The consensus results were analyzed in two ways. First, abnormality detection, comparing the classification performance between the normal DAT binding pattern and the other two categories (DP and nonDP). Second, DP detection, contrasting DP patterns against a combined group of nonDP and normal DAT binding.

Diagnostic consistency was assessed by calculating inter-rater agreement among the three evaluators and intra-rater agreement between real and generated delayed images separately for abnormality detection and DP detection in both the internal and independent validation sets. These results were then compared between real and generated delayed images.

### Statistical analysis

Continuous variables are presented as mean ± standard deviation, while categorical variables are presented as numbers. Pearson’s correlation coefficients were used to evaluate the correlation of striatal SNBRs between real and generated delayed images. Receiver operating characteristic (ROC) analysis was conducted to evaluate diagnostic performance based on quantitative metrics, and permutation tests were performed to compare ROC-AUC values. Consensus visual assessments by the three readers were used to compare diagnostic performance. Accuracy, sensitivity, and specificity, along with their respective 95% confidence intervals, were calculated to evaluate the ability of real and generated images to detect abnormalities or DP. The McNemar test was used to compare diagnostic performances. Inter-rater agreement among the three evaluators was assessed using Fleiss’ kappa, while intra-rater agreement between real and generated delayed images was evaluated using Cohen’s kappa. Z-tests were conducted to compare kappa values for abnormalities or DP detection. P < 0.05 was considered statistically significant. All analyses were performed using the Python Scipy package (v.3.8).

## Results

### Training and validation datasets of the generated model with representative images

Overall, 578 dual-phase [^18^F]-FP-CIT PET scans were acquired using the Biograph TruePoint 40 scanner between April and November 2018. After excluding 101 scans owing to patient motion or deviations from the early imaging time window (30 ± 5 min), 477 scans were included, 430 for model training (mean age 67.7 ± 10.6 years, 231 females) and 47 for internal validation (mean age 73.3 ± 7.3 years, 25 females; Table 1).

**Table 1:** Patient characteristics of datasets used for the training, internal, and independent validation of the cGAN framework.

	Training	Internal validation	Independent validation
**Number of participants**	430	47	52
**Age, years (SD)**	67.7 (10.6)	73.3 (7.3)	60.4 (10.7)
**Sex (M/F)**	199/231	22/25	23/29
**PET/CT**	Biograph TruePoint 40	Biograph TruePoint 40	Biograph Vision 600
**Clinical diagnosis**			
**Volunteer**			35
**Non-DP**	177	16	
**DP**	253	31	17

cGAN, conditional generative adversarial networks; SD, standard deviation; M, male; F, female; Non-DP, non-degenerative parkinsonism; DP, degenerative parkinsonism; PET/CT, positron emission tomography/computed tomography.

The independent validation set was prospectively collected between June and December 2022. Of 60 initially screened participants (patients and healthy volunteers), 6 individuals withdrew consent. The remaining 54 individuals underwent both early-phase and DAT imaging using the Biograph Vision 600 digital PET/CT system. Two additional participants were excluded for not meeting the early imaging time criteria, resulting in 52 participants included in the analysis (S2 Fig).

Based on follow-up clinical diagnoses, 31 of 47 patients in the internal validation set were diagnosed with DP, whereas 16 were classified as non-DP. In the independent validation set, 17 of 52 participants were confirmed to have DP, and 35 were healthy volunteers originally enrolled as controls (Table 1). Detailed patient characteristics and diagnostic categories for validation sets are provided in S2 Table.

**Fig 2:**
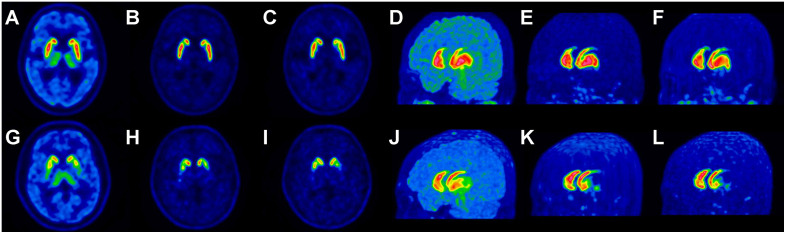
Two representative cases with transaxial and MIP images from paired early-phase, generated delayed-phase, and real delayed-phase PET scans in the internal validation set. **(a–f)**: Case 1: 73-year-old male with essential tremors showing no significant reduction in uptake in the bilateral striatum. **(g–l)**: Case 2: 40-year-old male with PD showing decreased DAT binding in the striatum, especially the PP. In each case, the transaxial and MIP images are arranged from left to right in the following order: early-phase PET (a, d, g, j), generated delayed-phase PET (b, e, h, k), and real delayed-phase PET (c, f, i, l). Abbreviations**:** MIP, maximum intensity projection; PET, positron emission tomography; PD, Parkinson’s disease; DAT, dopamine transporter; PP, posterior putamen.

**Fig 3:**
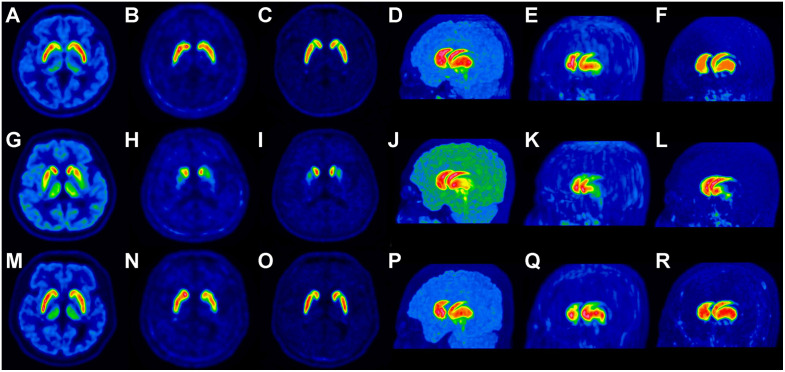
Three representative cases with transaxial and MIP images from paired early-phase, generated delayed-phase, and real delayed-phase PET scans in the independent validation set. **(a–f)**: Case 1: 41-year-old male (normal control) demonstrating no significant reduction in uptake in the bilateral striatum. However, the CA in the generated delayed image (b, e) exhibits a relatively shorter contour than the real delayed image. **(g–l)**: Case 2: 59-year-old male with PD revealing decreased DAT binding in the striatum, particularly in the PP, with a ventrodorsal gradient. **(m–r)**: Case 3: 56-year-old male (normal control) with discrepancies observed, particularly in the left CA. Visual analysis classified the generated image (n, q) as non-DP by consensus, while the real image (o, r) was correctly diagnosed with a normal DAT binding. In each case, the transaxial and MIP images are arranged from left to right in the following order: early-phase PET (a, d, g, j, m, p), generated delayed-phase PET (b, e, h, k, n, q), and real delayed-phase PET (c, f, i, l, o, r). Abbreviations: MIP, maximum intensity projection; PET, positron emission tomography; CA, caudate nucleus; PD, Parkinson’s disease; DAT, dopamine transporter; PP, posterior putamen; DP, degenerative parkinsonism.

Figures 2 and 3 present representative images of generated delayed-phase images with paired respective real delayed-phase images from an internal validation set using a conventional PET system (Biograph TruePoint 40) and an independent validation set using a digital PET system (Biograph Vision 600).

### Quantitative evaluation of image quality and diagnostic performance of the generated delayed images

The SSIM index between real and generated delayed-phase images demonstrated satisfactory results in the internal and independent validation sets with mean values of 94.92 ± 0.02 and 96.64 ± 0.01, respectively. The PSNR values were 33.73 ± 1.78 and 32.87 ± 0.87, while the RMSE values were 0.18 ± 0.02 and 0.22 ± 0.03, respectively (S3 Fig).

**Fig 4:**
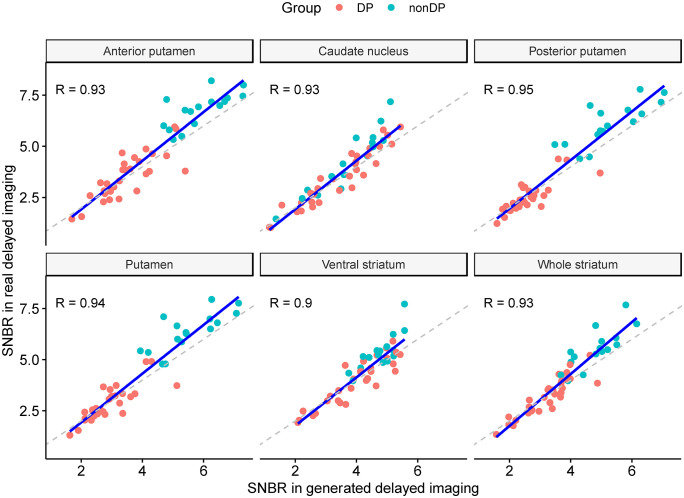
Scatter plots showing the correlation between SNBR obtained from generated and real delayed-phase PET images in the internal validation set. The SNBR of the whole striatum exhibited a high correlation, with an R-value of 0.93. Subregional SNBRs showed high correlations, ranging from 0.90 for the VS to 0.95 for the PP. Abbreviations: SNBR, specific to non-specific binding ratio; PET, positron emission tomography; VS, ventral striatum.

**Fig 5:**
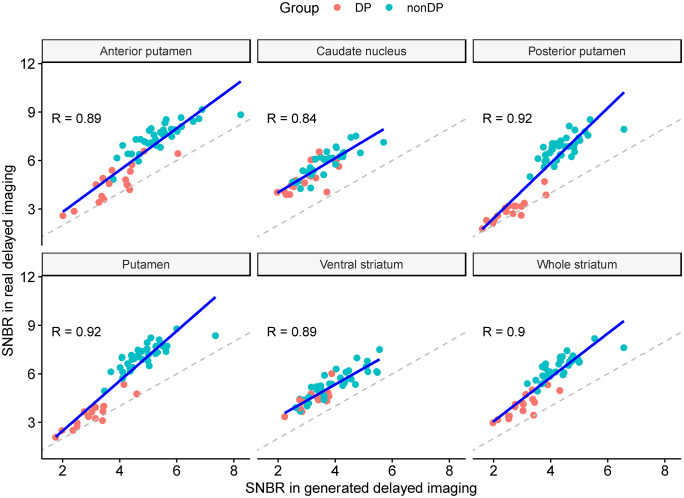
Scatter plots showing the correlation between SNBR from generated and real delayed-phase PET images in the independent validation set. The SNBR of the whole striatum exhibited a high correlation, with an R-value of 0.90. Subregional SNBRs exhibited high correlations, ranging from 0.84 for the CA to 0.92 for the PP. Abbreviations: SNBR, specific to non-specific binding ratio; PET, positron emission tomography; PP, posterior putamen. CA, caudate nucleus.

The SNBR values of the whole striatum showed strong correlations between real and generated delayed images, with R values of 0.93 and 0.90 in the internal and independent validation sets, respectively. Among subregions, the PP showed the highest correlation coefficients of 0.95 and 0.92. The lowest correlation coefficient was observed in the VS (0.90) for the internal validation set and CA (0.84) for the independent validation set (Figs 4 and 5).

ROC analysis with permutation tests regarding DP diagnosis using subregional SNBRs and ISR for both real and generated delayed images achieved the highest AUC values in the PP for the internal validation set. Significant differences in AUC values between real and generated delayed images were observed only in the two subregions with the lowest AUC values: VS and CA. For all other subregions and ISRs, including the whole striatum, no significant difference in diagnostic performance for DP was observed. A similar pattern was observed in the independent validation set, where both real and generated delayed images achieved the highest AUC values in the PP. Additionally, the lowest AUC values were observed in the VS and CA, except for PP/AP. No significant difference was observed in diagnostic performance among the subregional SNBRs and ISRs (Table 2).

**Table 2:** ROC-AUC with permutation tests regarding the diagnosis of DP using subregional SNBRs and ISRs in the internal and independent validation sets.

	Internal validation (Biograph Truepoint 40)			Independent validation (Vision 600)		
	**Generated**	**Real**	**p**	**Generated**	**Real**	**p**
**WS**	0.95	0.96	0.57	0.95	0.99	0.13
**P**	0.98	0.99	0.58	0.96	1.00	0.20
**AP**	0.97	0.99	0.26	0.90	0.97	0.07
**PP**	0.98	1.00	0.23	0.98	1.00	0.22
**CA**	0.57	0.64	0.02	0.76	0.81	0.31
**VS**	0.73	0.83	0.03	0.79	0.83	0.27
**CA/P**	0.87	0.90	0.24	0.97	1.00	0.16
**PP/AP**	0.87	0.89	0.61	0.91	0.99	0.04
**C/VS**	0.40	0.42	0.52	0.46	0.47	0.82
**P/VS**	0.95	0.98	0.52	0.83	0.94	0.82

ROC, receiver operating characteristic; AUC, area under the curve; DP, degenerative parkinsonism; SNBR, specific to non-specific binding ratio; ISR, inter-subregional ratio; WS, whole striatum; P, putamen; AP, anterior putamen; PP, posterior putamen; CA, caudate nucleus; VS, ventral striatum; CA/P, caudate nucleus to putamen ratio; PP/AP, posterior to anterior putamen ratio; C/VS, caudate nucleus to ventral striatum ratio; P/VS, putamen to ventral striatum ratio

### Visual analysis of generated delayed images

In the internal validation set, no images were rated as poor by any of the three readers based on noise and motion in real or generated delayed images. The proportion of images rated as good was comparable between real and generated delayed images across all readers (39/47 vs 38/47, 45/47 vs 42/47, 47/47 vs 47/47). When grouping good and fair ratings as interpretable and comparing them against poor (uninterpretable), no significant differences were observed. In the independent validation set, although inter-reader variability was noted in the image quality assessment, no images were rated as poor (uninterpretable). However, the proportion of images rated as good differed between real and generated delayed images (51/52 vs 15/52, 52/52 vs 5/52, 51/52 vs 50/52). Consistent with the internal validation set, grouping good and fair as interpretable showed no significant differences compared to that of poor (uninterpretable) ratings (S3 Table).

For the diagnostic performance based on visual assessment in the internal validation set, the accuracy, sensitivity, and specificity of generated delayed images for abnormality detection were slightly lower than those of real delayed images. However, these differences were not significant. Differences were even smaller for DP detection (Table 3). In the independent validation cohort, while the model showed comparable performance to real delayed images for DP detection, its accuracy for identifying general abnormalities was significantly lower than that of real delayed images (63.5% vs. 96.2%, p < 0.001). Specifically, subtle striatal defects in some non-degenerative cases were not fully recovered in the generated images. Sensitivity did not differ significantly between real and generated images for both abnormality and DP detections (Table 4).

**Table 3:** Diagnostic performance in detecting abnormalities or DP based on consensus interpretations of generated and real delayed-phase imaging in the internal validation set.

	Abnormality detection			Degenerative Parkinsonism detection		
	**Generated**	**Real**	**p**	**Generated**	**Real**	***p***
**Accuracy**	85.1% (72.3–92.6)	95.7% (85.8–98.8)	0.453	95.7% (85.8–98.8)	100.0% (92.4–100.0)	1
**Sensitivity**	96.9% (84.3–99.4)	100.0% (89.3–100.0)	1	96.8% (83.8–99.4)	100.0% (89.0–100.0)	1
**Specificity**	60.0% (35.7–80.2)	86.7% (62.1–96.3)	0.219	93.8% (71.7–98.9)	100.0% (80.6–100.0)	1
**TN**	9	13		15	16	
**FP**	6	2		1	0	
**FN**	1	0		1	0	
**TP**	31	32		30	31	

Data presented with 95% confidence interval; DP, degenerative parkinsonism; TN, true negative; FP, false positive; FN, false negative; TP, true positive.

**Table 4:** Diagnostic performance in detecting abnormalities or DP based on consensus interpretations of generated and real delayed-phase imaging in the independent validation set.

	Abnormality detection				Degenerative Parkinsonism detection	
	**Generated**	**Real**	**p**	**Generated**	**Real**	***p***
**Accuracy**	63.5% (49.9–75.2)	96.2% (87.0–98.9)	2E-05	94.2% (84.4–98.0)	100.0% (93.1–100.0)	0.25
**Sensitivity**	100.0% (81.6–100.0)	100.0% (81.6–100.0)	NA	100.0% (81.6–100.0)	100.0% (81.6–100.0)	NA
**Specificity**	45.7% (30.5–61.8)	94.3% (81.4–98.4)	2E-05	91.4% (77.6–97.0)	100.0% (90.1–100.0)	0.25
**TN**	16	33		32	35	
**FP**	19	2		3	0	
**FN**	0	0		0	0	
**TP**	17	17		17	17	

Data presented with 95% confidence interval; DP, degenerative parkinsonism; TN, true negative; FP, false positive; FN, false negative; TP, true positive; NA, not applicable

Inter-reader and intra-reader agreements were assessed for both abnormality and DP detection. In the internal validation set, inter-reader agreement was slightly higher for real delayed images than for generated delayed images in abnormality detection (0.86 vs 0.79, p = 0.29), with a similar trend observed for DP detection (0.97 vs 0.88, p = 0.1232; S4 Table). Intra-reader agreement between real and generated delayed images was higher for DP detection compared to that of abnormality detection (Table 5).

**Table 5:** Intra-reader agreement between generated and real delayed-phase images for detecting abnormalities or DP in the internal and independent validation sets.

	Internal validation (Biograph Truepoint 40)				Independent validation (Vision 600)			
	**Original 3 class**	**Abnormality detection**	**DP detection**	**p**	**Original 3 class**	**Abnormality detection**	**DP detection**	**p**
Intra-reader1	0.62	0.58	0.86	0.0227	0.45	0.38	0.76	0.0081
Intra-reader2	0.7	0.67	0.86	0.0842	0.81	0.88	0.87	0.5211
Intra-reader3	0.76	0.73	0.95	0.0202	0.33	0.2	0.8	0.0001
Consensus	0.62	0.6	0.91	0.011	0.48	0.41	0.87	0.0006

DP, degenerative parkinsonism

In the independent validation set, inter-reader agreement for abnormality detection was significantly higher for real delayed images than for generated delayed images (0.97 vs 0.38, p < 0.0001). However, inter-reader agreement for DP detection remained slightly higher for real delayed images (1.00 vs 0.82, p = 0.0114; S4 Table). Intra-reader agreement between real and generated delayed images was higher or comparable for DP detection than for abnormality detection (Table 5).

## Discussion

This study demonstrated that deep learning-based generated delayed [^18^F]-FP-CIT PET images achieved comparable diagnostic performance to those of real delayed images, particularly for detecting DP. This suggests the potential clinical utility of AI-generated images in minimizing patient discomfort and radiation exposure without significantly compromising diagnostic accuracy [27,28].

Despite the high structural similarity between generated and real delayed images, certain differences were observed, particularly in CA uptake and striatal contour. This aligns with previous studies reporting that deep learning models may introduce subtle regional variations owing to factors such as limited training data, image noise, or model optimization constraints [29,30]. The reduced CA uptake in generated images corresponded with the quantitative results, where Pearson’s correlation coefficient for this region was slightly lower than that of other striatal subregions. Prior studies similarly report that deep learning-generated medical images may inadequately reproduce fine structural details, potentially explaining the variations observed in this study [31,32].

From a visual assessment perspective, nuclear medicine specialists accurately interpreted most of the generated images, with comparable interpretability between real and generated images across both the internal and independent validation cohorts. However, image quality ratings differed significantly between the two datasets, with generated images in the independent validation set less frequently rated as “good”. This suggests that differences in PET scanner types (analogue vs. digital) potentially affect model generalizability, an issue previously noted in the literature regarding AI applications within nuclear imaging [33–35]. Future studies should consider incorporating more diverse training datasets to enhance model robustness across various imaging platforms.

Our results showed that while the cGAN model maintained high diagnostic accuracy, there was a statistically significant difference in SNBR correlation in the independent validation set compared to the internal set. This can be attributed to the ‘domain gap’ between conventional and digital PET systems. The Biograph Vision 600 provides superior contrast recovery and spatial resolution, which the model trained on lower-resolution TruePoint 40 data may perceive as an out-of-distribution feature.

Despite these minor discrepancies, the diagnostic performance of the generated delayed images remained robust. ROC curve analysis revealed no significant differences in AUC values for diagnosing DP between real and generated images across most subregions, except for the VS and CA in the internal validation set and the PP/AP ratio in the independent validation set. These findings suggest that although generated images are effective for disease detection, subtle regional differences in uptake values may persist and warrant further investigation [30,36].

The diagnostic performance of our model should be interpreted within the context of the class balance in our study cohorts. The prevalence of DP was higher in the training and internal validation sets, which facilitated the model’s ability to learn and identify subtle DAT loss patterns. However, the independent validation set comprised a higher proportion of healthy volunteers. As noted in the Results, this shift in class balance, combined with the presence of non-specific artifacts in digital PET imaging, likely contributed to the observed increase in false-positive findings and the subsequent decrease in general abnormality detection accuracy.

Inter-reader and intra-reader agreement analyses further support the clinical feasibility of this approach. Although inter-reader agreement was slightly lower for generated images in abnormality detection, it remained high for DP detection. This indicates that although nuclear medicine specialists might recognize subtle differences in generated images, these variations do not significantly affect diagnostic decision-making.

Our study demonstrated a slight decrease in diagnostic accuracy in the independent validation set obtained from a digital PET system. This discrepancy likely reflects a domain shift arising from differences in detector technology and system characteristics between scanner generations. Recent studies suggest that deep learning based harmonization methods may further mitigate such cross scanner variability [37]. From the perspective of recent generative modeling, different architectures present distinct trade-offs. Variational autoencoders (VAEs) typically provide stable training and fast inference but often produce overly smooth outputs with limited high frequency detail. Diffusion based models [38], such as denoising diffusion probabilistic models (DDPMs), can achieve high synthesis fidelity and improved distribution matching but generally require substantially longer iterative inference. Generative adversarial networks (GANs), including the conditional GAN (cGAN) framework used in this study, provide a practical balance between image fidelity and computational efficiency. Therefore, the cGAN-based approach used in this study represents a reasonable compromise for clinical application, where both image quality and inference speed are important considerations for (near) real-time workflows.

The multi-slice 2.5D cGAN architecture represents a compromise between spatial consistency and computational/sample-size feasibility. While fully volumetric 3D models may further improve inter-slice continuity and global coherence, they require substantially greater GPU memory and larger training datasets to avoid overfitting. Although computationally efficient, patch-based approaches may limit the ability to capture global tracer redistribution patterns across the full field of view. This limitation is particularly relevant for synthesizing delayed-phase PET images from early-phase data, where uptake changes may occur over relatively large anatomical regions.

A key finding of this study is the performance gap in detecting general abnormalities within the independent cohort. This limitation likely arises from the heterogeneity of non-DP abnormalities, such as vascular parkinsonism or drug-induced cases, which were underrepresented in the training phase. Since cGAN models tend to generate images that follow the dominant distribution of the training data, they may ‘regularize’ or ‘smooth over’ atypical, patchy uptake patterns often seen in non-degenerative parkinsonism. For instance, Case 3 in Fig 3 illustrates a scenario where a subtle caudate-dominant defect was misinterpreted by the model as a normal variant. Therefore, while our model is highly effective for screening DP, clinicians should exercise caution and rely on real delayed-phase imaging when subtle, non-typical DAT distribution is suspected.

In this study, we used empirically selected preprocessing methods. We found that SUVR 10 was sufficient for representing intact normal striatal uptake in delay-phase PET images. Therefore, we used 10 as the upper normalization limit and scaled SUVR [0,10] to the aforementioned normalized range of [−1, 1] for labelling the generator network. This count normalization was effective in controlling SUVR dynamic range variability across normal and abnormal DAT binding of participants.

This study has several clinical implications. Generating reliable delayed [^18^F]-FP-CIT PET images from early-phase scans could streamline imaging workflows by shortening patient examination times and alleviating logistical challenges in high-volume clinical settings. This approach is potentially advantageous in resource-limited environments where prolonged imaging sessions may be restricted. Additionally, minimizing patient wait times and radiation exposure aligns with ongoing efforts to enhance patient safety and comfort in nuclear medicine procedures [39].

This study had some limitations. First, although the deep learning model demonstrated high performance, it was trained on a relatively homogeneous dataset. Future research should incorporate larger, more diverse cohorts to evaluate model generalizability across varied populations and PET scanner types. Second, while diagnostic performance was the primary focus, the clinical effect of implementing generated images in real-world practice requires further investigation. Prospective, multicenter studies involving diverse nuclear medicine specialists are crucial to validate these findings. To enhance clinical generalizability across different institutions, future iterations of the model should incorporate multi-scanner training data or utilize unsupervised domain adaptation (UDA) techniques. Such approaches would allow the model to ‘harmonize’ features from various acquisition settings without requiring paired datasets from every new scanner type, thereby facilitating broader clinical adoption in centers with diverse PET hardware. Finally, exploring alternative deep learning architectures, such as transformer-based models, may further enhance image fidelity and diagnostic accuracy [40,41].

## Conclusions

In conclusion, our cGAN-based model demonstrated the feasibility of generating synthetic delayed-phase [^18^F]-FP-CIT PET images from early-phase scans. While the AI-generated images showed excellent diagnostic performance and strong quantitative correlation for detecting degenerative parkinsonism (DP), they were less sensitive in identifying general, non-degenerative abnormalities, particularly in independent validation using a digital PET system. These results suggest that while the proposed AI technique can effectively streamline clinical workflows for PD screening, caution is warranted when interpreting cases with subtle or non-typical DAT patterns. Future studies incorporating more diverse pathological cohorts are needed to further enhance the model’s generalizability and diagnostic breadth.

## Supporting information

S1 Fig Schematic of the cGAN model synthesizing delayed-phase PET images from early-phase inputs. cGAN, conditional generative adversarial network; PET, positron emission tomography.
(DOCX)

S2 Fig Enrollment flow chart of the prospective cohort for the independent validation. PD, Parkinson’s disease; PET, positron emission tomography.
(DOCX)

S3 Fig Box plots showing evaluation metrics—SSIM, PSNR, and RMSE—for both the internal and independent validation sets. SSIM, structural similarity index measure; PSNR, peak signal-to-noise ratio; RMSE, root mean square error.
(DOCX)

S1 Table Comparison of the acquisition protocols and reconstruction parameters for conventional and digital PET/CT system. (DOCX)

S2 Table Detailed patient characteristics and diagnostic categories for validation sets. (DOCX)

S3 Table Image quality assessments by three readers for generated and real delayed-phase images in the internal and independent validation sets. (DOCX)

S4 Table Inter-reader agreements for generated and real delayed-phase images in detecting abnormalities or DP in the internal and independent validation sets. (DOCX)
