# Supplementary material for: Development of AI-based dopamine transporter (DAT) image generation technique using early phase [18F]-FP-CIT PET imaging
Source: PLoS One. 2026 May 14;21(5):e0349375. doi: 10.1371/journal.pone.0349375 (PMC13175495; doi:10.1371/journal.pone.0349375)
Supplement: S1 Fig — cGAN, conditional generative adversarial network; PET, positron emission tomography. (DOCX) [file pone.0349375.s001.docx]

**
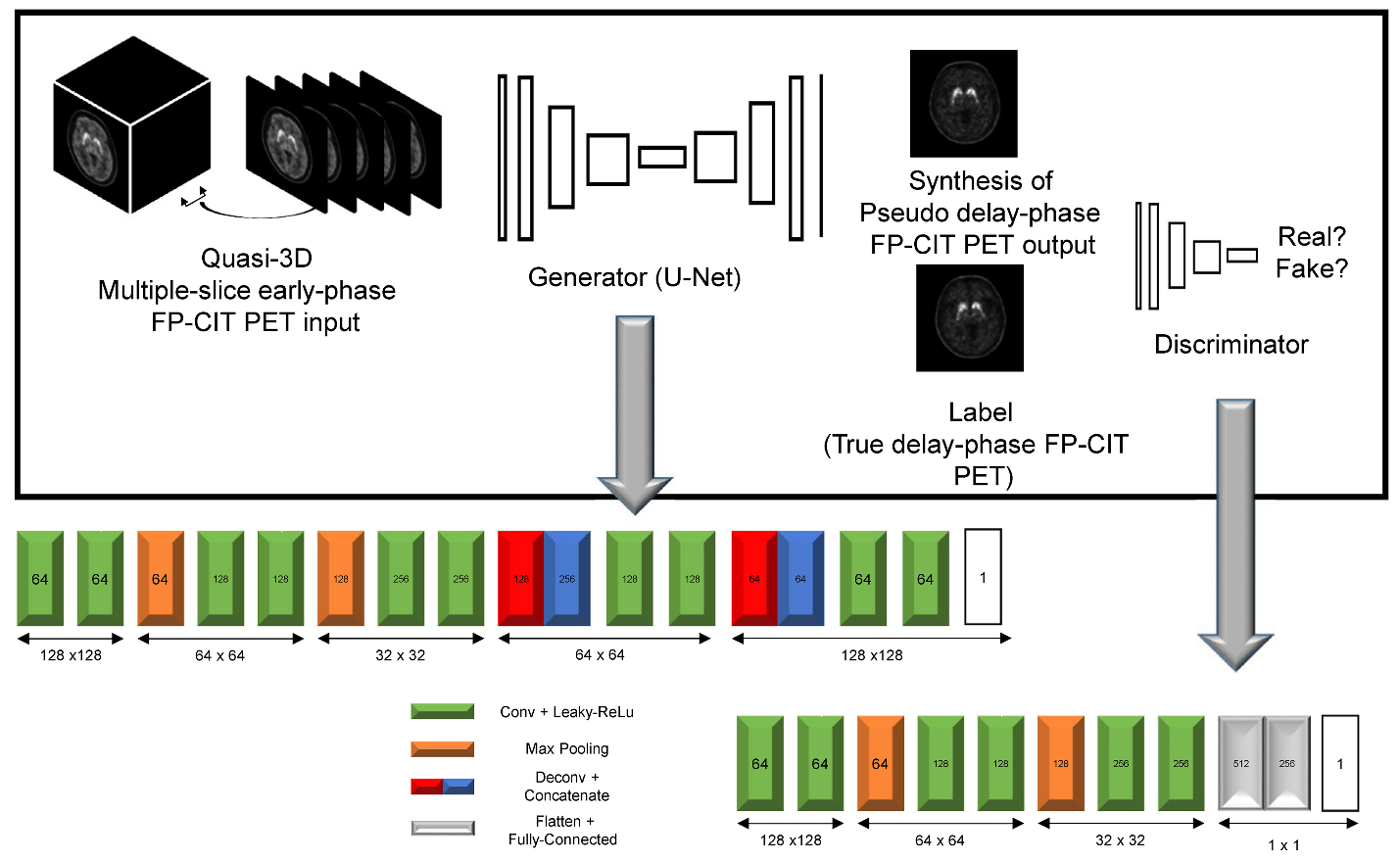
**

**S1 Fig**. **Schematic of the cGAN model synthesizing delayed-phase PET images from early-phase inputs.**

cGAN, conditional generative adversarial network; PET, positron emission tomography.
