# Supplementary material for: Development of AI-based dopamine transporter (DAT) image generation technique using early phase [18F]-FP-CIT PET imaging
Source: PLoS One. 2026 May 14;21(5):e0349375. doi: 10.1371/journal.pone.0349375 (PMC13175495; doi:10.1371/journal.pone.0349375)
Supplement: S2 Fig — PD, Parkinson’s disease; PET, positron emission tomography. (DOCX) [file pone.0349375.s002.docx]

**S2 Fig**. **Enrollment flow chart of the prospective cohort for the independent validation**

PD, Parkinson’s disease; PET, positron emission tomography.
