# Supplementary material for: Development of AI-based dopamine transporter (DAT) image generation technique using early phase [18F]-FP-CIT PET imaging
Source: PLoS One. 2026 May 14;21(5):e0349375. doi: 10.1371/journal.pone.0349375 (PMC13175495; doi:10.1371/journal.pone.0349375)
Supplement: S3 Fig — SSIM, structural similarity index measure; PSNR, peak signal-to-noise ratio; RMSE, root mean square error. (DOCX) [file pone.0349375.s003.docx]

**S3 Fig**. **Box plots showing evaluation metrics—SSIM, PSNR, and RMSE—for both the internal and independent validation sets**. SSIM, structural similarity index measure; PSNR, peak signal-to-noise ratio; RMSE, root mean square error.
