# Supplementary material for: Development of AI-based dopamine transporter (DAT) image generation technique using early phase [18F]-FP-CIT PET imaging
Source: PLoS One. 2026 May 14;21(5):e0349375. doi: 10.1371/journal.pone.0349375 (PMC13175495; doi:10.1371/journal.pone.0349375)
Supplement: S1 Table — (DOCX) [file pone.0349375.s004.docx]

**S1 Table. Comparison of the acquisition protocols and reconstruction parameters for conventional and digital PET/CT system**

| Parameter | Conventional PET/CT | Digital PET/CT |
| --- | --- | --- |
| Scanner Model | Biograph Truepoint 40 | Biograph Vision 600 |
| Detector Technology | PMT-based | SiPM-based |
| In-plane Resolution (FWHM) | 2.0 mm | < 2.0 mm |
| Time-of-Flight (TOF) | Not applied | Applied |
| Reconstruction Algorithm | OSEM + PSF (TrueX) | OSEM + PSF (TrueX) + TOF |
| Iterations / Subsets | 6 iterations / 16 subsets | 8 iterations / 5 subsets |
| Post-smoothing Filter | All-pass (None) | 2.0 mm Gaussian |
| Matrix Size | 336 × 336 | 440 × 440 |
| Voxel Size (mm³) | 0.89 × 0.89 × 1.5 | 0.68 × 0.68 × 1.5 |
| Acquisition Window (Early) | 30–40 min p.i. | 30–40 min p.i. |
| Acquisition Window (Delayed) | 180 min p.i. | 180 min p.i. |

PET/CT, positron emission tomography/computed tomography; FWHM, full width at half maximum; PMT, photomultiplier tube; SiPM, silicon photomultiplier; TOF, time-of-flight; OSEM, ordered subset expectation maximization; PSF, point spread function; p.i., post-injection.
