# Supplementary material for: Development of AI-based dopamine transporter (DAT) image generation technique using early phase [18F]-FP-CIT PET imaging
Source: PLoS One. 2026 May 14;21(5):e0349375. doi: 10.1371/journal.pone.0349375 (PMC13175495; doi:10.1371/journal.pone.0349375)
Supplement: S2 Table — (DOCX) [file pone.0349375.s005.docx]

**S2 Table. Detailed patient characteristics and diagnostic categories for validation sets**

| Characteristics | Internal Validation (n=47) | Independent Validation (n=52) |
| --- | --- | --- |
| Degenerative Parkinsonism (DP) | 31 (66.0%) | 17 (32.7%) |
| - Parkinson's Disease (PD) | 28 (59.6%) | 17 (32.7%) |
| - Atypical Parkinsonism | 3 (6.4%) |  |
| - H&Y Stage (Mean ± SD) | 2.18 ± 0.77 | 2.11 ± 0.52 |
| - Disease duration (Month) | 24.1 ± 30.8 | 48.4 ± 50.7 |
| Non-Degenerative (Non-DP) | 16 (34.0%) | 35 (67.3%) |
| - Vascular parkinsonism | 1 (2.1%) |  |
| - Normal / Essential Tremor | 15 (31.9%) | 35 (67.3%) |
| - Disease duration (Month) | 47.2 ± 74.7 |  |
| Total Class Balance (DP:Non-DP) | 31 : 16 | 17 : 35 |

Atypical Parkinsonism : 2 MSA, 1 CBD; Normal / Essential Tremor : 10 ET, 1 AD, 1 AD with ET, 1 Blepharospasm, 2 Normal subjects
