# Supplementary material for: Development of AI-based dopamine transporter (DAT) image generation technique using early phase [18F]-FP-CIT PET imaging
Source: PLoS One. 2026 May 14;21(5):e0349375. doi: 10.1371/journal.pone.0349375 (PMC13175495; doi:10.1371/journal.pone.0349375)
Supplement: S3 Table — (DOCX) [file pone.0349375.s006.docx]

| **S3 Table. Image quality assessments by three readers for generated and real delayed-phase images in the internal and independent validation sets** | | | | | | | |
| --- | --- | --- | --- | --- | --- | --- | --- |
|  | **Reader 1** | | | **Reader 2** | | **Reader 3** | |
|  | | **Generated** | **Real** | **Generated** | **Real** | **Generated** | **Real** |
| **Internal** | |  |  |  |  |  |  |
| **Good** | | 38 | 39 | 42 | 45 | 47 | 47 |
| **Fair** | | 9 | 8 | 5 | 2 | 0 | 0 |
| **Poor** | | 0 | 0 | 0 | 0 | 0 | 0 |
| **Independent** | |  |  |  |  |  |  |
| **Good** | | 15 | 51 | 5 | 52 | 50 | 51 |
| **Fair** | | 37 | 1 | 47 | 0 | 2 | 1 |
| **Poor** | | 0 | 0 | 0 | 0 | 0 | 0 |
