# Supplementary material for: Development of AI-based dopamine transporter (DAT) image generation technique using early phase [18F]-FP-CIT PET imaging
Source: PLoS One. 2026 May 14;21(5):e0349375. doi: 10.1371/journal.pone.0349375 (PMC13175495; doi:10.1371/journal.pone.0349375)
Supplement: S4 Table — (DOCX) [file pone.0349375.s007.docx]

| **S4 Table. Inter-reader agreements for generated and real delayed-phase images in detecting abnormalities or DP in the internal and independent validation sets** | | | | | | |
| --- | --- | --- | --- | --- | --- | --- |
| **Datasets** | **Internal validation** | | | **Independent validation** | | |
| **Fleiss' kappa** | **Generated** | **Real** | ***p*** | **Generated** | **Real** | ***p*** |
| **Original 3 class** | 0.78 | 0.86 | 0.2638 | 0.51 | 0.97 | 0.0001 |
| **Abnormality detection** | 0.79 | 0.86 | 0.29 | 0.38 | 0.97 | < 0.0001 |
| **DP detection** | 0.88 | 0.97 | 0.1232 | 0.82 | 1 | 0.0114 |
| DP, degenerative parkinsonism | |  |  |  |  |  |
